# High type II error and interpretation inconsistencies when attempting to refute transgenerational epigenetic inheritance

**DOI:** 10.1186/s13059-016-0982-4

**Published:** 2016-07-12

**Authors:** Carlos Guerrero-Bosagna

**Affiliations:** Avian Behavioral Genomics and Physiology Group, IFM Biology, Linköping University, Linköping, 58 183 Sweden

## Abstract

A recently published article in *Genome Biology* attempts to refute important aspects of the phenomenon of transgenerational epigenetic inheritance (TEI). An alternative explanation of the data is offered here, showing that TEI is indeed not contradicted.

Please see related Correspondence article: www.dx.doi.org/10.1186/s13059-016-0981-5 and related Research article: http://genomebiology.biomedcentral.com/articles/10.1186/s13059-015-0619-z

A growing number of publications report the existence of transgenerational epigenetic inheritance (TEI). TEI is based on environmentally-induced epigenomic changes in the germ line that affect the somatic and/or germ line epigenomes of individuals in subsequent generations, as well as their phenotypes [1]. Evidence of TEI is available since the first report in 2005 [2] and has emerged from a variety of model organisms, including rodents, fish, and invertebrates [3]. Also, reports of disease phenotypes being transgenerationally transmitted in humans [4] make TEI of wide interest for current and future human health [5].

Due to the relatively recent description of the phenomenon of TEI and the complexity of the molecular mechanisms involved, it is not surprising that many knowledge gaps remain. The group of Dr. Szabó recently published a study in *Genome Biology* [6] conducted in mice and well designed to address some major questions in the process of TEI. These include “how are environmentally-induced germ line epigenomic changes maintained in subsequent generations?” and “how do environmentally-induced epigenomic changes observed in the mature sperm correlate with epigenomic marks in fetal germ cells?” Pregnant mice were exposed to environmental toxicants previously shown to induce TEI (e.g. BPA [7–10], DHEP [11], and vinclozolin [2, 8, 12, 13]). Germ line DNA methylation was then assessed in the immediate offspring (G1) and their descendants (G2). Based on their data, the authors’ main conclusion was that there is no evidence for TEI at the level of germ line DNA methylation because changes in DNA methylation “are not found in the germ cells of the subsequent generation.”

The present correspondence aims at offering an alternative explanation of the data presented by Iqbal et al. [6], in order to clarify that no data in that paper contradicts current evidence on the process of TEI. Upon careful reading of the article, it is apparent that the main conclusions are not supported by the results. Moreover, the results indeed provide evidence for TEI. Other authors have recently criticized aspects of the manuscript [6] that are not covered in this correspondence [14, 15]). Here, important methodological issues are discussed such as: (1) the high type II error observed, which relates to the low number of animals used in the DNA methylation comparison (2 controls versus 2 treatments); and (2) the inconsistency between the data shown and the conclusions drawn.

## Number of individuals used for comparisons

The number of individual samples used in the study [6] (not shown in the “Methods” section, but only in the legends of Figure 3 and Additional file 10) indicates “n = 3” (possibly meaning 3 controls versus 3 treatments) for fetal male germ cells (MGC) comparisons and “n = 2” (possibly meaning 2 controls versus 2 treatments) for sperm comparisons. The MIRA-chip signals of the 2 versus 2 (sperm) or 3 versus 3 (MGC) comparisons are shown in their Figure 8 and Additional figure 9. One important consequence of using such low numbers of individuals for comparisons is that it does not allow for a powerful enough statistical testing in order to detect differences among groups, leading to a substantial increase in type II error.

A post hoc power analysis was performed with the ssize R package [16], employing an average of the standard deviations provided by Dr. Szabó and the same FDR rate (0.05) used for power calculations by her group. The results are shown in Table 1.

**Table 1:** Power analysis for 2 vs. 2 comparisons using the ssize R script

Fold change	Power (%)	Type II error (%)
1.2	9.44	90.56
1.3	10.96	89.04
1.4	12.57	87.43
1.5	14.27	85.73
1.6	16.05	83.95
1.7	17.91	82.09
1.8	19.83	80.17
1.9	21.81	78.19
2	23.85	76.15

The main conclusion from this table is that the 2 versus 2 comparisons are under-powered to detect even twofold changes. In such cases, the type II error gives a 76.6 % chance of not detecting changes. This level is far from the accepted standard, which is a ~20 % chance of not finding effects. Therefore, all the analyses that include sperm samples, i.e. those performing 2 versus 2 comparisons, are under-powered to detect either small (20 %) or large (100 %) changes in DNA methylation.

As for the 3 versus 3 comparisons, Table 1 in Dr. Szabó’s response shows that in order to detect a 20 % change in methylation (a fairly common rate of change), 14 individuals would have been needed in order to obtain a power of 0.8. My conclusion is that although the 3 versus 3 comparisons seem sufficient to detect a 50 % change in methylation, they are also under-powered to detect common changes such as 20 %.

Another consequence of studying a small number of individuals is specifically related to epigenetic analyses. Epigenetic variation exists between animals so that variability in methylation patterns will occur among individuals in the same gene. When a population of animals is affected by an environmental stimulus, not all individuals will be affected to the same extent, similarly to what occurs for physiological parameters in response to environmental disturbances.

Variability in DNA methylation changes observed among individuals can only be detected when a sufficient number of individuals is studied. In a 2 versus 2 animal testing design (as used in [6]), there is an enormous chance that non-responsive or less-responsive individuals are compared, leading to the erroneous conclusion that no change occurs due to the treatment.

Sufficient statistical power is especially important in studies that aim at refuting previous findings. Based on the current data evaluation, my conclusion is that the DNA methylation analyses presented in the Iqbal et al. study [6] does not have sufficient power to refute the aspects of TEI in question. The fact that few genes were found altered is strongly dependent on the low power of the experiments. Moreover, it is also important to consider that even if a few changes are found, they can still be of biological relevance.

## Inconsistencies between the results and the conclusions drawn

Interestingly, even with the low number of individuals used for the comparisons, important differences were detected representing transgenerational transmission of germ line epigenetic changes. These, however, are not apparent due to the way the data are presented in their Table 3.

**Fig. 1:**
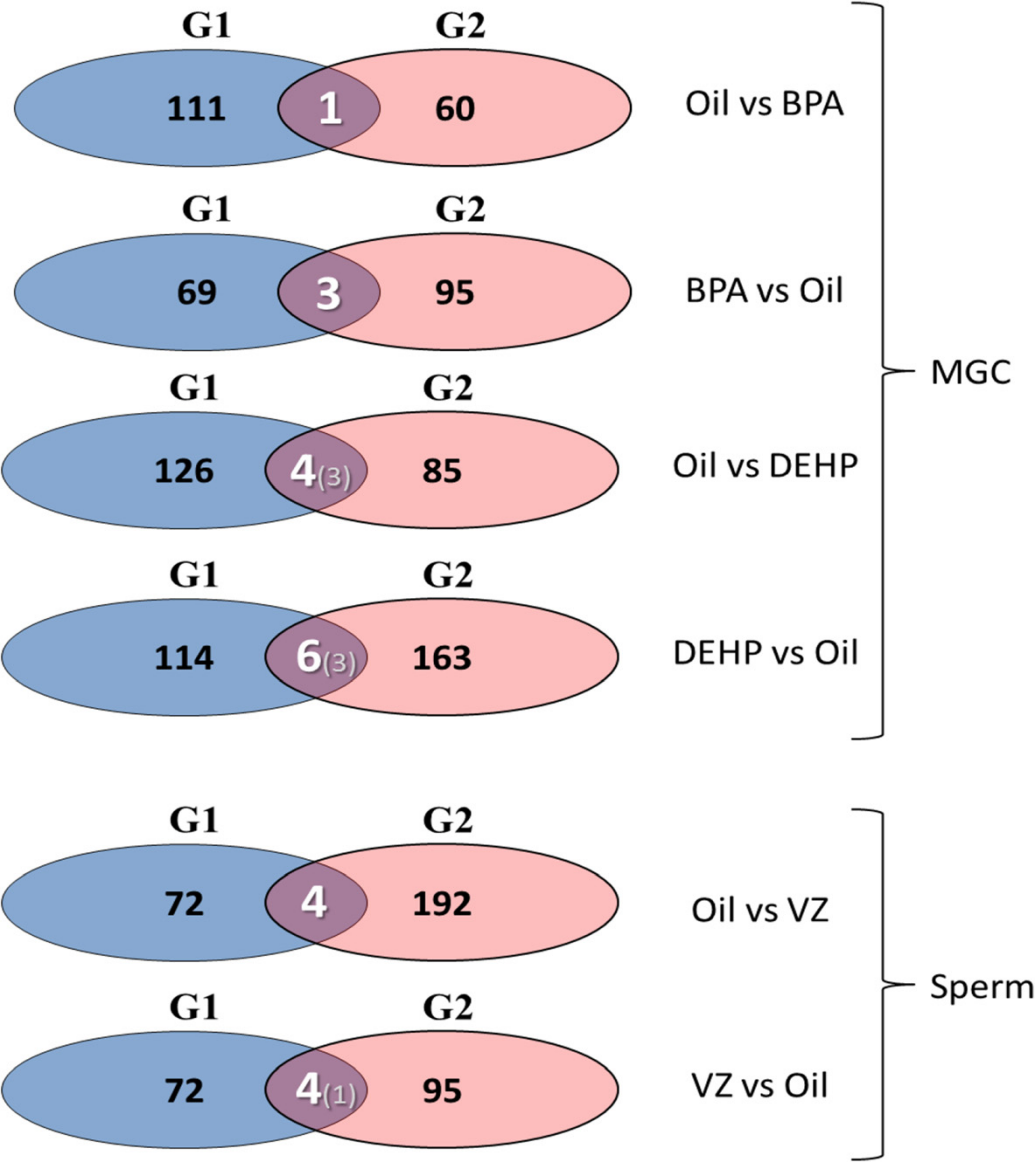
*Venn diagram representations* built using the same data shown in Table 3 of Iqbal et al.’s study [6]. Numbers inside the *balloons* represent the genes with altered DNA methylation in each generation (G1 or G2), in sperm or MGC, in response to each exposure tested (BPA, DEHP, or vinclozolin). The *intersections* between the G1 and G2 generation balloons show the number of common genes epigenetically altered in these two generations in response to the different exposures

Venn diagram representations shown here (Fig. 1) are built using the same data shown in their Table 3 [6]. The intersection between the G1 and G2 generation balloons shows the number of common genes epigenetically altered in these two generations in response to the different exposures. The genes altered with the same direction of methylation change in both generations are shown in parenthesis.

**Fig. 2:**
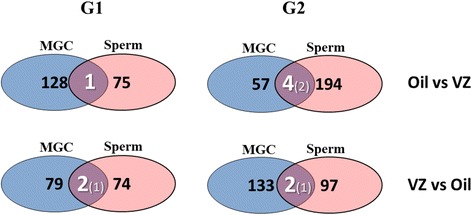
*Venn diagram representations* built using the same data shown in Table 3 of Iqbal et al.’s study [6]. Numbers inside the *balloons* represent the genes with altered DNA methylation in each generation (G1 or G2), in sperm or MGC, in response to vinclozolin exposure. The *intersection* between the “MGC” and “Sperm” balloons shows the number of common genes epigenetically altered in these two differentiation stages, in each generation. DNA methylation alterations in exactly the same direction in “MGC” and “Sperm” are shown in parenthesis

Figure 2 shows a similar comparison but focuses on the common genes altered in DNA methylation between MGC and testis, for each generation.

These data clearly show numerous genes altered in the germ line in both generations tested, with some of them being common between them. Furthermore, these changes are observed with all the exposures tested. In my opinion, these results, together with the high type II error derived from the low number of individuals tested, do not support the main conclusions of the Iqbal et al. paper [6] regarding not finding evidence for TEI in their study. Even though the majority of the changes observed are not the same in each generation, they are still indicative of a transgenerational epigenomic effect in the germ line.

## About our previous research in TEI

A very last point relates to Dr. Szabó’s criticisms of our previous work regarding sample pooling and reproducibility of our findings. In regards to pooling samples, this is a very common and statistically valid method to account for biological variability in procedures of high cost [17, 18]. By pooling, the values obtained are equivalent to arithmetical averages [17, 18]. With regards to the reproducibility, this was so paramount in our studies that candidate genes were selected only after they have appeared as significantly changed in all three of the comparative hybridization comparisons performed. Moreover, afterwards candidates were tested with other local DNA methylation techniques such as pyrosequencing, bisulphite sequencing, or MeDIP-qPCR [12, 19]. A candidate gene was called true only after passing all these filters.

Type II error acquires special relevance in studies attempting to refute previous findings. Even though Dr. Szabó criticizes the power in our previous studies (which indeed included a higher number of individuals within the pooled samples), our main aim was not to refute previous findings, rather to provide reliable evidence for transgenerational changes in DNA methylation. As described above, we reported genes after they have passed many layers of confirmation, which makes the statistical possibility that these are “random effects” very low. However, underpowered experiments will probably not detect these differences, since most of them are below 20 % changes in DNA methylation. With regards to the relevance of small changes in DNA methylation, it is up to the reader to evaluate their biological importance. In my personal opinion, the biological effects of such changes in DNA methylation, especially when combined gene actions are considered, should not be overlooked.
